# Polyelectrolyte Polysaccharide–Gelatin Complexes: Rheology and Structure

**DOI:** 10.3390/polym12020266

**Published:** 2020-01-26

**Authors:** Svetlana R. Derkach, Yuliya A. Kuchina, Daria S. Kolotova, Nikolay G. Voron’ko

**Affiliations:** Department of Chemistry, Murmansk State Technical University, Sportivnaya str., 13, Murmansk 183010, Russia; uak2008@mail.ru (Y.A.K.); kolotovads@gmail.com (D.S.K.); voronkonikolay@mail.ru (N.G.V.)

**Keywords:** gelatin, polysaccharide, chitosan, κ-carrageenan, sodium alginate, polyelectrolyte complexes, gelatin secondary structure, rheological properties

## Abstract

General features of rheological properties and structural peculiarities of polyelectrolyte polysaccharide–gelatin complexes were discussed in this paper. Experimental results were obtained for typical complexes, such as κ-carrageenan–gelatin, chitosan–gelatin and sodium alginate–gelatin complexes. A rheological method allows us to examine the physical state of a complex in aqueous phase and the kinetics of the sol–gel transition and temperature dependences of properties as a result of structural changes. The storage modulus below the gelation temperature is constant, which is a reflection of the solid-like state of a material. The gels of these complexes are usually viscoplastic media. The quantitative values of the rheological parameters depend on the ratio of the components in the complexes. The formation of the structure as a result of strong interactions of the components in the complexes was confirmed by UV and FTIR data and SEM analysis. Interaction with polysaccharides causes a change in the secondary structure of gelatin, i.e., the content of triple helices in an α-chain increases. The joint analysis of the structural and rheological characteristics suggests that the formation of additional junctions in the complex gel network results in increases in elasticity and hardening compared with those of the native gelatin.

## 1. Introduction

Different types of interactions between bioactive macromolecules are of considerable and possibly decisive importance in numerous areas, such as studies of processes in nature and living organisms, food industry [1,2,3] and medical technologies [4,5,6]. The formation and disintegration of polysaccharide–protein macromolecular complexes are typical examples of such interactions, depending on compositions, environment, temperature and so on. There are many publications devoted to these subjects, and it would be unreasonable to try listing numerous original publications, especially since there are several comprehensive reviews covering different aspects of the issue [7,8,9,10]. However, dominant problems discussed in these reviews involve chemistry, thermodynamics and kinetics of polyelectrolyte formation. This is very important and interesting, but the understanding of mechanical and particularly rheological properties of polysaccharide–protein complexes, especially in relation to their structures, is also extremely important for applications. However, this aspect has not been considered in much detail. 

This publication is devoted mainly to the results of studies on rheological properties of polysaccharide–protein complexes in correlation with the analysis of their structures. The discussion is based on experimental data related to gelatin as the most popular representative of proteins and κ-carrageenans, which is a typical polysaccharide, as a co-gelator, although experimental data for some other polysaccharides are also used.

## 2. Materials and Methods

An alkaline grade gelatin (G) Type B from bovine skin (225 g), Bloom, (Sigma-Aldrich, St. Louis, MO, USA) was used. Its isoelectric point determined using viscometric and turbidimetric methods was equal to 4.7.

The sample of κ-carrageenan (Car) (Sigma-Aldrich, Søborg, Denmark) used in experiments (*M*_η_ = 430 kDa) had a moisture content of 7.7 wt %. The chitosan (Chit) sample (Sigma–Aldrich, Reykjavik, Iceland) was obtained from shrimp shells; the degree of deacetylation was 86% for *M*_η_ = 260 kDa. A sample of sodium alginate (alginic acid sodium salt, SA) (Sigma-Aldrich, Dorset, United Kingdom) from brown algae with *M*_η_ = 630 kDa was used. 

Solutions of polysaccharides (P) and gelatin were prepared separately and dissolved at 70 and 40 °C, respectively. Aqueous mixtures of polysaccharide–gelatin complexes were prepared by mixing stock solutions at 40 °C. Chitosan was dissolved in 0.1 M acetic acid at 70 °C after preliminary swelling for one day at 20 °C. Mixtures of biopolymers with a constant concentration of gelatin, C_G_, in a range of 1.0 to 5.0 wt % and different concentrations of polysaccharide were prepared. Thus, polysaccharide/gelatin (*w*/*w*) ratios, Z = C_P_/C_G_, varied from 0.001 to 1.00 in the mixtures. The pH values of the mixtures of gelatin with κ-carrageenan and sodium alginate were in a range of 5.2–5.8. The pH values of chitosan and its mixtures with gelatin were in a range of 3.1–3.9. One can find the details of characterisation of the samples used in [11,12,13]. 

The main method used for studying the structural transformation was FTIR analysis. The FTIR spectra of the samples were obtained using an FTIR spectrometer (Shimadzu FTIR Tracer-100 and Shimadzu IRAffinity-I, Kyoto, Japan) at a 4 cm^−1^ resolution by accumulating 256 scans in a range of 3600–800 cm^−1^ [14,15]. The experiments were performed as following: dried films of samples were grinded in a ball mill until it was in the state of a highly dispersed powder, and then, this powder was pressed with KBr to form a pellet. In order to minimize the effect of traces of moisture (water vapour), the spectrum for water vapour was subtracted from the spectra obtained.

Moreover, the microstructure was examined by SEM (S405-A Hitachi, Tokyo, Japan) using the SEM LEO-420 software. Before measurements, hydrogels formed by polysaccharide–gelatin complexes were kept in a desiccator at a temperature of 12 °C for 4 days. Then, the samples were dried using a setup that avoided deformation and destruction of the samples during drying under a high vacuum, and a conductive gold layer with a thickness range of 1–20 nm was sprayed on these samples by vacuum deposition.

Rheological properties of all samples were measured in the shear deformation mode using a rheometer (Physica MCR301, Anton Paar, Graz, Austria) with a cone-and-plate working unit (the diameter of the cone was 50 mm, and the angle between the conical surface and the plate was 1 grad). Measurements were carried out in the following deformation modes:

- periodic oscillations at a constant temperature (14 °C) and a different frequency, ω, in a range of 0.0628–628 rad/s in the domain of linear viscoelastic behaviour;
- at a constant temperature of 14 °C and a constant frequency of 6.28 rad/s to follow the “aging” of the samples;
- at a constant frequency of 6.28 rad/s and increasing the temperature at a rate of 2 K/min;
- in rate-controlled shearing mode with a range of shear rates, $\dot{\gamma }$, of 10^−3^–10^2^ s^−1^ or in stress with the yield stress σ control mode in a range of 0.01–200 Pa.

The variation of a given temperature was within ± 0.1 °C. The reproducibility of the rheological results was controlled by parallel testing of two samples with the same content.

## 3. Results

At temperatures below 30 °C, gelatin macromolecules undergo a coil/triple helix (ordered form) conformational transition. Such a transition is accompanied by a decrease in the solubility of gelatin at cooling, the formation of intermolecular contacts and the formation of a three-dimensional (3D) polymer network, if the concentration of gelatin exceeds some critical thresholds [16,17]. As a result, the gelatin sol transforms into a gel; the gelation process proceeds in time.

**Figure 1:**
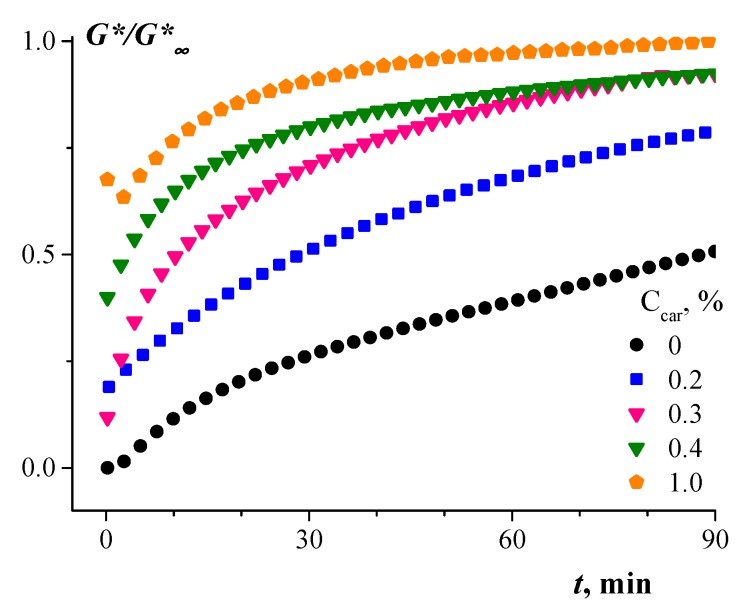
Kinetics of gelation represented by the complex dynamic modulus, G*, normalised by its limiting value, G*_∞_. The concentration of gelatin was 2 wt %. The concentrations of κ-carrageenan, C_car_, are shown.

The gelation properties of the κ-carrageenan–gelatin polyelectrolyte complexes were monitored as they changed with time, because the kinetics of gel formation was of primary interest. The method of measuring the evolution of rheological properties of a material is rather convenient and simple. Figure 1 presents the results of the isothermal evolution of the relative changes in the complex elastic modulus (current value of G* divided by its final limiting values G*_∞_ reached as results of long experiments). 

A representative treatment of experimental data can be achieved by presenting the time dependence of the relative change of the elastic modulus, which can be written as:

$$\tilde{G}=\frac{G*\left(t\right)-G_{0}^{*}}{G_{\infty }^{*}-G_{0}^{*}}.$$

The dependence, $\tilde{G}\left(t\right)$, is a kinetic curve that can be fitted by the equation of the first order:

$$\tilde{G}=1-ke^{-t/\theta },$$

where *k* is the fitting constant and *θ* is the characteristic time.

**Figure 2:**
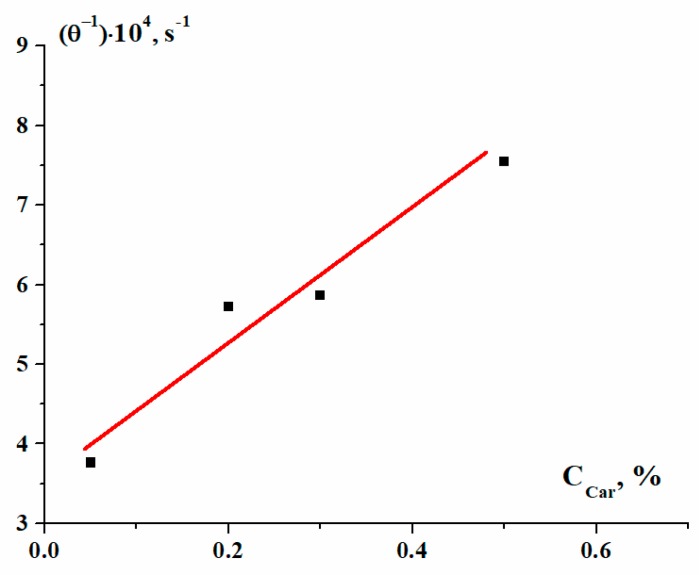
Dependence of the characteristic constant rate of gel formation on the concentration of polysaccharide.

The constant rate (*θ*^−1^) of gel formation depended on the concentration of κ-carrageenan, C_car_, and this dependence is shown in Figure 2.

Evidently, increasing the κ-carrageenan concentration results in the acceleration of the complex gel formation.

Therefore, we can write the kinetic equation as:

$$\frac{d\tilde{G}}{\tilde{G}dt}=K\left(1-\tilde{G}\right)\left[C_{car}\right],$$

where *K* is a constant. This is a typical first-order kinetic equation with a constant *K*. An increase in gelation rate was also found, for example, with increases in alginate-to-gelatin [18] and gellan-to-gelatin [19] ratios.

It is well-known that gelatin macromolecules can pass through a transition at approximately 20–27 °C with coil/helix transition [16,17]. In our case, this was accompanied by a sol/gel transition that occurred due to the formation of intermolecular hydrogen bonds between carboxyl oxygen and amide hydrogen in the polypeptide chain. This transition is of a kinetic character, which is typical for soft matter. One of the most popular methods is detecting the crossover point, where the temperature dependencies of components of a complex dynamic modulus (measured at some constant frequencies) become equal to each other. This is conditionally assumed in a gel state of the storage modulus (G′) over the loss modulus (G′′) and in a sol state of G′ < G′′. Then, the equality of G′(T) = G′′(T) corresponds to the transition temperature, T*.

**Figure 3:**
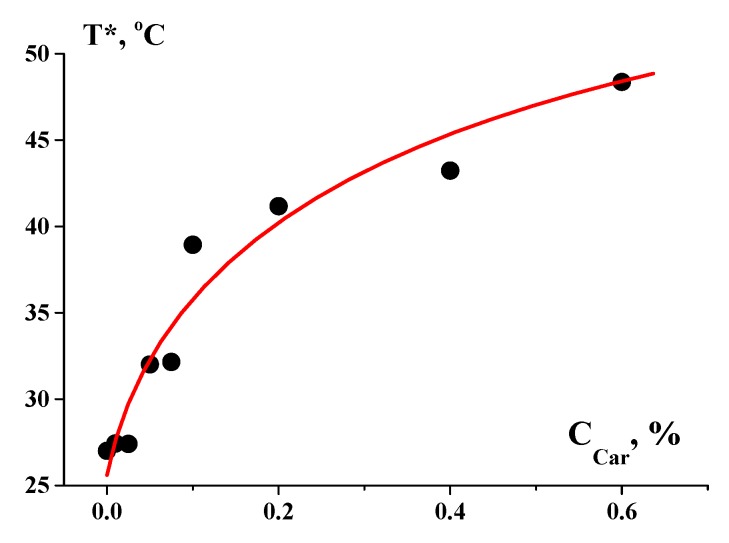
Temperature dependence of the gel/sol transition of gelatin (C_G_ = 1 wt %) on the concentration of complex polysaccharides.

The addition of polysaccharides promotes increase in the rigidity of the molecular structure [19,20] and thereby the preservation of the helix conformation of a complex, increasing the transition temperature. Experimental data illustrating the dependence of T* defined by this method on the *κ*-carrageenan concentration, C_Car_, are shown in Figure 3. The addition of gellan increases the melting temperature and the mechanical strength of a fish gelatin film [21]. In some cases, the thermal stability of gelatin films decreases with an increase in chitosan content [22].

**Figure 4:**
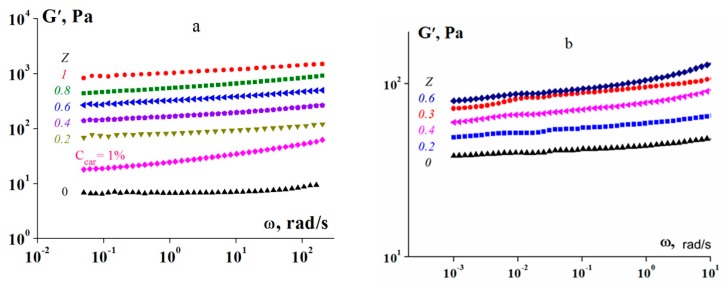
Frequency dependences of storage modulus *G*′ for the κ-carrageenan–gelatin (**a**) and chitosan–gelatin (**b**) complexes at a gelatin concentration C_G_ of 1 wt % and for different component mass ratios, Z (indicated in the graph).

The viscoelastic properties of all complex gels below T* are characteristic of solid-like soft materials. This frequency independence of the storage modulus *G*′(ω) dominates the storage modulus over the loss modulus (*G*′(ω) > *G*′′(ω)), although the existence of losses reflects the possibility of relaxation processes in the material. Several examples of experimental data of *G*′(ω) are presented in Figure 4, where data regarding *G*′′(ω) are omitted. The synergistic effect of polysaccharides in physical gelatin gels increases the storage modulus [18,23].

The temperature, T*, is a certain threshold that divides the two domains with different rheological properties. At *T* > T*, solutions of polyelectrolyte complexes are fluids (maybe with non-Newtonian properties), because the temperature destroys the labile secondary bonds, which can create a supramolecular structure. Then, even at low stresses, solutions can flow. Meanwhile, the solutions of polyelectrolyte complexes at *T* < T* can flow at high stresses to demonstrate viscoelastic behaviour, which means that a 3D structure in such solutions is created by secondary bonds with a much higher strength rather than at higher temperatures. The strength of this structure is quantitatively characterised by the yield stress, σ_Y_.

The rheological properties of polyelectrolyte complexes in the temperature range above T* are characterised by flow curves—shear rate or shear stress dependencies of apparent viscosity. Indeed, at *T* < T*, practically in all cases, these flow curves are typical for viscoplastic liquids. This is typical for emulsions and concentrated suspensions that are soft matter [24,25].

**Figure 5:**
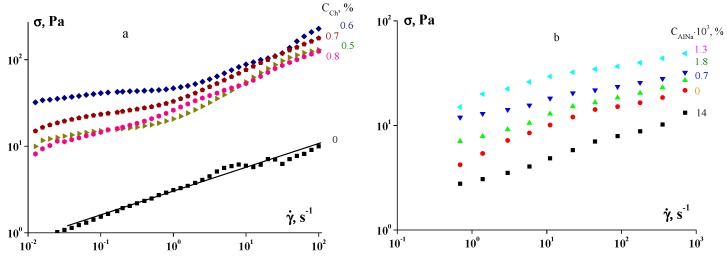
Viscous properties of native gelatin (the concentration of gelatin is 1 wt %) and complexes of chitosan (**a**) and sodium alginate (**b**) with gelatin. The concentrations of polysaccharide are shown.

Some examples of flow curves of several gels of polyelectrolyte complexes are presented in Figure 5.

**Figure 6:**
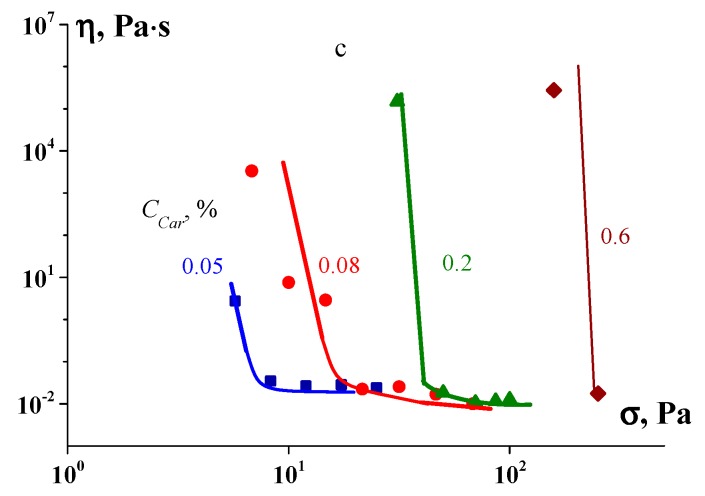
Yields in complexes of native gelatin (the concentration of gelatin is 1 wt %) with *κ*-carrageenan. The concentrations of *κ*-carrageenan used are shown.

Meanwhile, at low stresses, one can observe a typical yielding behaviour with the yield stress dependent on the concentration of the polysaccharide (Figure 6).

**Figure 7:**
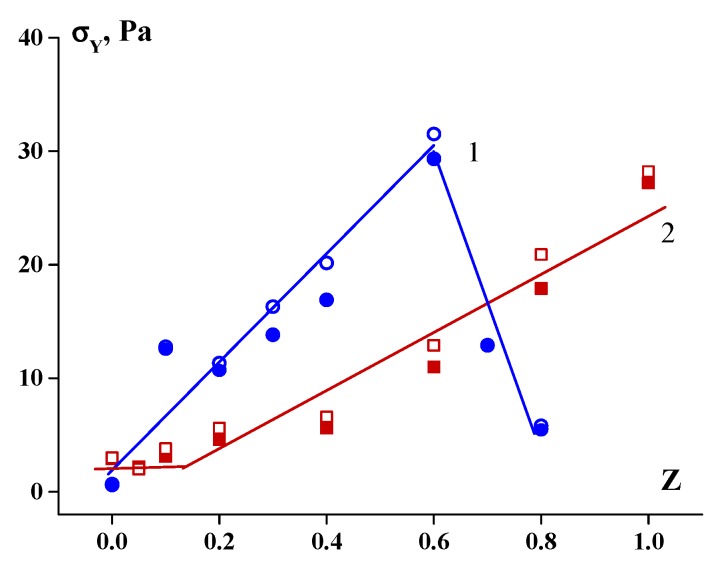
Yield stress σ_Y_ as a function of the polysaccharide/gelatin mass ratio, Z, in the complexes. Polysaccharides: 1—chitosan; 2—κ-carrageenan. The σ_Y_ values are determined by the Hershel–Bulkley (open points) and Casson (full points) models. C_G_ = 1 wt %.

The flow curves of rather dilute solutions of individual components (either polysaccharides or gelatin) are Newtonian or slightly non-Newtonian liquids, and only the formation of complexes leads to the creation of a structure characterised by the appearance of a clearly noticeable yield stress. Thus, the most expressive effect can be expected, if we follow the dependence of the yield stress, σ_Y_, on the ratio, Z, of the concentration of the added polysaccharides to the concentration of the base gelatin in a common system. As mentioned above, defining the yield stress is ambiguous, depending on the chosen method (like the determination of T*). The usual method is based on fitting experimental data by an empirical equation, e.g., by the Bingham, Casson or Hershel–Bulkley equations, where σ_Y_ is used as a fitting parameter. Several examples shown in Figure 7 illustrate the influence of the complex formation on the value of the yield stress as determined using one of these equations.

In some cases, σ_Y_ begins to abruptly grow at some critical value of *Z*, and this threshold value Z* corresponds to the formation of complexes, whereas in other cases, the dependence of σ_Y_(*Z*) passes through a maximum at some Z* value. The nonmonotonic dependence of the yield stress indicates certainly the influence of the complex composition on its gel-forming properties and the strength of the gel structure [26].

Actually, rheological data are very expressive, but indirect evidence of the polyelectrolyte complex formation is the consequence of this phenomenon. Therefore, it is important to correlate the rheological data with direct chemical arguments, which can be followed by FTIR spectra. 

Intermolecular interactions between the polysaccharide and gelatin leading to the formation of polyelectrolyte complexes were confirmed by UV and FTIR spectroscopy data. 

**Figure 8:**
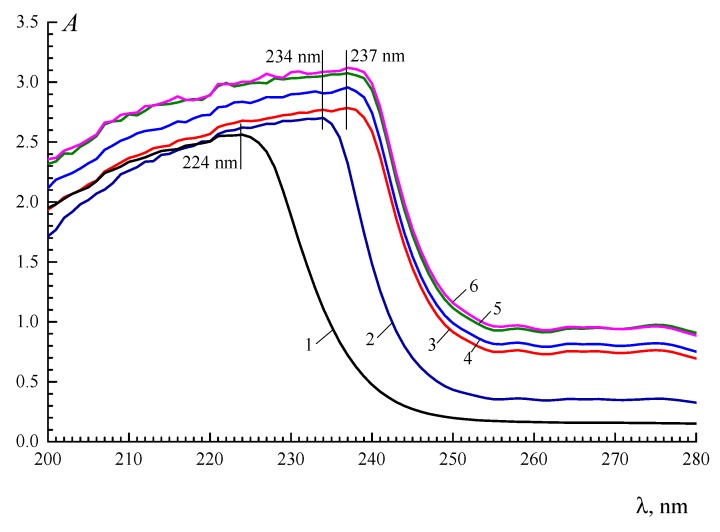
UV absorption spectra of chitosan sol (1), gelatin sol (2) and chitosan–gelatin mixtures (3–6). C_G_ = 1.0 wt %; polysaccharide-to-gelatin mass ratio, Z: (3) 0.2, (4) 0.3, (5) 0.4, and (6) 0.5.

Figure 8 shows, as an example, the UV absorption spectra of chitosan sol, gelatin sol and their mixtures with different mass ratios, *Z*. The influence of polysaccharides on the spectra is not additive. The interaction of polysaccharides with gelatin results in a bathochromic shift of the maximum absorption, regardless of the chitosan content. This red shift of the gelatin spectrum indicates the electrostatic interaction between amino groups in chitosan and typical chromophores, such as carboxylic groups, Glu and Asp, which absorb radiation in the near-UV range [14]. An increase in absorption in the structureless absorption band (see the right-hand parts of the spectra of the mixtures) can be reasonably ascribed to light scattering by relatively large particles of the complexes. An increase in absorption in this spectral region and a red shift of the absorption maximum in the gelatin UV spectra were also observed for mixtures with κ-carrageenan [11] and sodium alginate [13].

**Figure 9:**
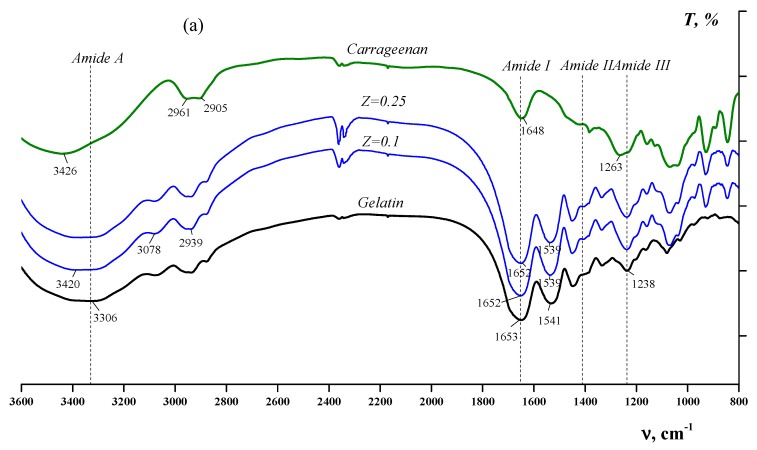

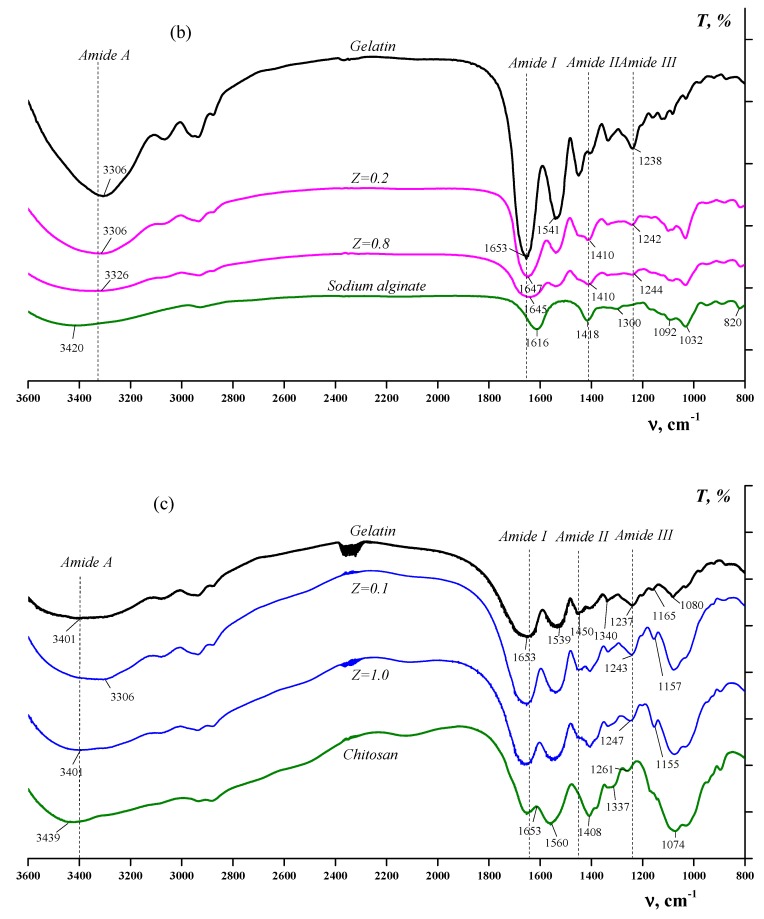
FTIR spectra of gelatin, polysaccharides and complexes of κ-carrageenan (**a**), sodium alginate (**b**) and chitosan (**c**) with gelatin at different polysaccharide-to-gelatin (*w*/*w*) ratios, Z.

An analysis of the FTIR spectra of the mixtures at various ratios, Z (Figure 9), compared with the FTIR spectra of individual components and characteristic absorption bands (Table 1) [27,28,29], allows us to distinguish the type of interactions that occurs.

The introduction of polysaccharides into a gelatin solution (Figure 9a–c) leads to a shift of the characteristic band of gelatin amide A to the high-frequency region (in the case of κ-carrageenan or alginate) or to the low-frequency region (in the case of chitosan), which is explained by the formation of hydrogen bonds between biopolymer macromolecules [30,31]. The blue shift can also be explained by electrostatic interactions between the carboxyl groups of an alginate or sulphate group of κ-carrageenan with amide groups (Arg, Lys, Hyl and His) of gelatin during the formation of complexes [32,33].

Moreover, the addition of polysaccharides causes a shift of the amide I band (Table 1) in native gelatin spectra to lower frequencies (Figure 9a,b). The red shift indicates electrostatic interactions of positively charged amide groups of gelatin with negatively charged carboxyl groups of sodium alginate or sulphate groups of κ-carrageenan. Similar red shifts were observed for alginate–gelatin membranes [34] and films [33], as well as for gellan–gelatin complexes [21]. The proof of electrostatic interactions between negatively charged carboxyl groups in gelatin and positively charged amino groups in chitosan is the shift of the 1165 cm^−1^ band of gelatin towards the low-frequency range (see Figure 9c). The same effect was observed in [35].

The observed spectral alterations in the FTIR spectra of native gelatin confirmed strong intermolecular interactions (electrostatic interactions and hydrogen bonding) between polypeptide chains of gelatin and macromolecules of polysaccharides during the self-organisation of polyelectrolyte complexes, which were also observed for complexes of gelatin with gellan [21,36], xanthan gum [37], agarose [38] and fully deacetylated chitosan [39].

**Figure 10:**
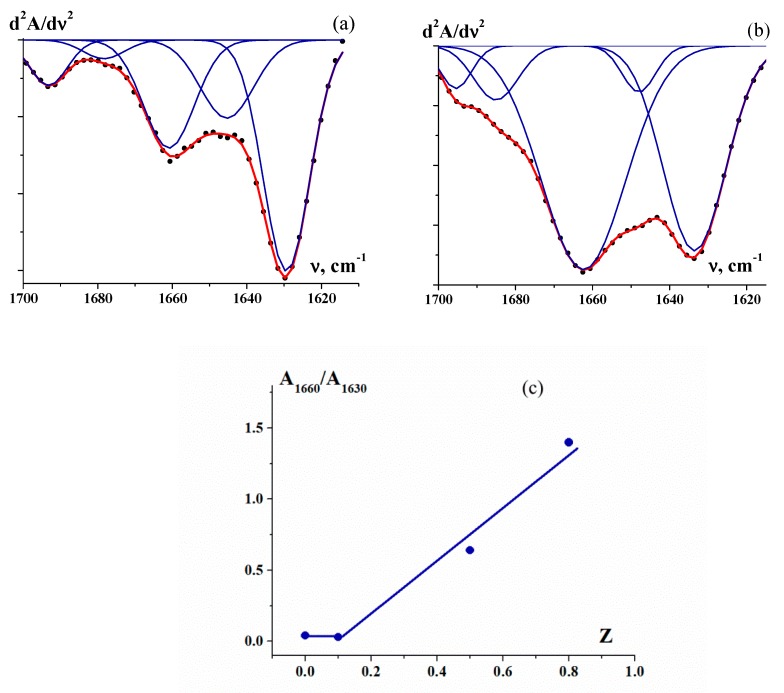
Decompositions of second-derivative FTIR spectra using five Gaussian components: (**a**) gelatin gel (C_G_ = 1 wt %); (**b**) κ-carrageenan–gelatin gels (polysaccharide-to-gelatin (*w*/*w*) ratio *Z* = 0.8); (**c**) the ratio of the areas of two bands (A_1660_/A_1630_) in the second-derivative spectrum of κ-carrageenan–gelatin complex gels. Decompositions were performed according to the procedure described in [44].

Strong intermolecular interactions cause a change in the secondary structure of gelatin, i.e., a change in the content of collagen-like triple-helix segments of an α-chain. Amide I is the band that is most sensitive to changes in the secondary structure of a protein [40,41,42]. Some examples of decomposition (The decomposition was carried out by O.N. Makshakova in the Laboratory of Biophysical Chemistry of Nanosystems at the Kazan Institute of Biochemistry and Biophysics, FRC Kazan Scientific Center of RAS.) are shown in second-derivative FTIR spectra in the amide I region (for gelatin and polyelectrolyte complexes), in which five Gaussian components appeared as a result of the absorbance of gelatin chains in different conformations, as presented in Figure 10. According to the literature [27,43], main components (bonds) at 1630, 1645, 1660, 1683 and 1692 cm^–1^ were assigned to β-sheets, random coils, triple helices, β-turn/b-sheets and β-turns, respectively.

The main bands at 1660 and 1630 cm^−1^ that display oppositely directed changes in the secondary structure were used to evaluate the relative variation in the triple helix content. As the polysaccharide/gelatin mass ratio, *Z*, increases, the secondary structure of the gelatin changes significantly. The ratio of the areas of two main bands (A_1660_/A_1630_) in the second-derivative spectrum of the κ-carrageenan–gelatin complexes shows a sharp increase above a threshold value, Z* (Z* = 0.1) (Figure 10c). Upon the formation of polyelectrolyte complexes with κ-carrageenan, gelatin chains obtain additional helicity that exceeds the triple helix content in the native gelatin. A similar effect was observed for complexation with sodium alginate [13].

In contrast, the analysis of the amide I region in the FTIR spectra of the chitosan–gelatin complexes and native gelatin (Figure 9b) demonstrated that the relative content of gelatin triple helices decreases at large *Z* values. Furthermore, the high-frequency shifts of amide III at *Z* = 0.8 and 1.0 show (according to [45,46]) a reduction in intermolecular interactions between gelatin chains within the collagen-like triple-helix structure. This effect could also be attributed to the decrease in the gelatin helix content. 

The evolution of the secondary structure of gelatin due to the formation of a complex with a polysaccharide is considered to be the cause of the change in the rheological behaviour of the complexes. Thus, an increase in the fraction of ordered structures (triple helices) of a gelatin chain (see Figure 10c) leads to the appearance of additional intermolecular contacts and, as a result, the elastic–viscous characteristics of gels formed by the polysaccharide–gelatin complexes increase (see Figure 4c and Figure 7). Similarly, chitosan–gelatin systems at high component ratios show that a decrease in the fraction of gelatin-ordered structures (FTIR spectral data) is accompanied by decreases in the viscoelastic parameters and strength (see Figure 7) of the gel of the complex.

**Figure 11:**
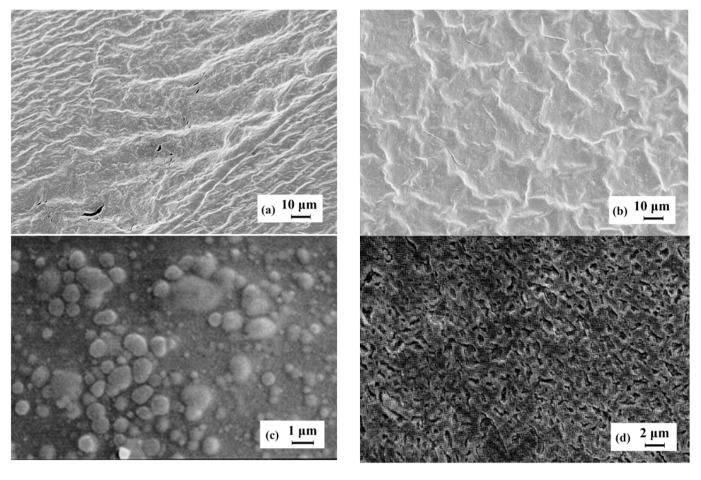
SEM images of the native gelatin hydrogel (**a**) and hydrogel formed by polyelectrolyte complexes with κ-carrageenan (**b**); sodium alginate (**c**) and chitosan (**d**) at *Z* = 0.03–0.05. Magnification: ×10^4^.

Changes in the secondary structure of the gelatin during the formation of complexes with a polysaccharide were confirmed by SEM (Figure 11). The SEM image (Figure 11a) confirms the supramolecular structure of the native gelatin formed by encountering collagen-like fibrils with a 3D network. The observed discrete zones (cells) located along the fibrils may be formed by uncoiled sections of macromolecules. 

Polysaccharide additives cause essential changes in a network structure (Figure 11b–d). The polysaccharide initiates some interactions between triple-helix zones in the gelatin owing to the appearance of polyelectrolyte complexes. In some cases (in the presence of sodium alginate, see Figure 11b), this may lead to the formation of complex aggregates. These polyelectrolyte complexes can play a role of additional nodes in a network [47,48] which is seen in Figure 11a,b and, therefore, lead to hardening of the supramolecular structure [20,49,50].

The effect of polysaccharide–gelatin polyelectrolyte complexes was observed at micro- and macrolevels; it is associated with hardening of the gelatin gel structure, which is evident in the rheological observations.

## 4. Conclusions

This study of polyelectrolyte complexes with different polysaccharides shows that they demonstrate similar rheological and structural effects owing to strong interactions between active groups in both components. The crucial influence on the structures and properties of these complexes is determined by the sol-to-solid-like (or sol–gel) transition. This transition, as well as the kinetics of the complex gel formation, was followed by a rheological method, and the obtained results correlated well with the evolution of UV and FTIR spectra and SEM images. From a rheological point of view, the sol-to-gel transition was reflected by the appearance of the solid-like structure. The latter was characterised by the frequency independence of the storage modulus (in the linear domain of viscoelasticity) and the viscoplastic flow behaviour. The strength of the structure created in the gel of the polyelectrolyte complexes, which was reflected by the yield stress, depended on the ratio of the components in the complexes. 

A strong interaction of the components and direct evidence of the supramolecular structural transformation were confirmed by UV (aqueous solutions) and FTIR (dried samples) spectral studies. In a certain concentration range, polysaccharides cause an increase in the helicity and, accordingly, rigidity of gelatin α-chains. The formation of additional network nodes containing gelatin triple helices interacting with polysaccharide macromolecular chains may cause the evolution of rheological properties, which involves increases in the elasticity and strength of the gel formed by the complexes.

## Figures and Tables

**Table 1:** Characteristic absorption bands of protein and polysaccharides.

Component	Wave Number ν (cm^−1^)	Type of Vibration
Gelatin	3400–3300	Stretching vibrations of NH groups (amide A)
	1700–1600	Stretching vibrations of CO– and CN– groups (amide I)
	1575–1480	Deformation vibrations of NH– groups and stretching vibrations of CN groups (amide II)
	1300–1230	Stretching vibrations of CN groups (amide III)
Sodium alginate	3600–3200	Stretching vibration of OH groups
	1580–1620	Asymmetric stretches of COO groups
	1400–1420	Symmetric stretches of COO groups
	1300–1320	Stretching vibration of CO groups
	1070–1090	Mannuronic units
	1025–1035	Guluronic units
	815–820	α-configuration of guluronic units
*k*-carrageenan	3600–3200	Stretching vibration of OH groups
	1270–1230	Vibration of the sulfate group
	1100–1080	Glycosidic bonds
	1070, 928–933	3,6-anhydridegalactose group
	840–850	D-galactose-4-sulfate group
Chitosan	3600–3200	Stretching vibrations of NH groups and OH groups
	2960–2880	Symmetric and asymmetric stretches of CH groups
	1670-1620	Stretching vibrations of CO groups (amide I)
	1325-1320	Stretching vibrations of CN groups (amide III)
